# SMMF: a self-attention-based multi-parametric MRI feature fusion framework for the diagnosis of bladder cancer grading

**DOI:** 10.3389/fonc.2024.1337186

**Published:** 2024-03-07

**Authors:** Tingting Tao, Ying Chen, Yunyun Shang, Jianfeng He, Jingang Hao

**Affiliations:** ^1^ Faculty of Information Engineering and Automation, Kunming University of Science and Technology, Kunming, China; ^2^ Department of Radiology, Second Affiliated Hospital of Kunming Medical University, Kunming, China; ^3^ School of Physics and Electronic Engineering, Yuxi Normal University, Yuxi, China

**Keywords:** bladder cancer, MP-MRI, deep learning, self-attention, feature fusion

## Abstract

**Background:**

Multi-parametric magnetic resonance imaging (MP-MRI) may provide comprehensive information for graded diagnosis of bladder cancer (BCa). Nevertheless, existing methods ignore the complex correlation between these MRI sequences, failing to provide adequate information. Therefore, the main objective of this study is to enhance feature fusion and extract comprehensive features from MP-MRI using deep learning methods to achieve an accurate diagnosis of BCa grading.

**Methods:**

In this study, a self-attention-based MP-MRI feature fusion framework (SMMF) is proposed to enhance the performance of the model by extracting and fusing features of both T2-weighted imaging (T2WI) and dynamic contrast-enhanced imaging (DCE) sequences. A new multiscale attention (MA) model is designed to embed into the neural network (CNN) end to further extract rich features from T2WI and DCE. Finally, a self-attention feature fusion strategy (SAFF) was used to effectively capture and fuse the common and complementary features of patients’ MP-MRIs.

**Results:**

In a clinically collected sample of 138 BCa patients, the SMMF network demonstrated superior performance compared to the existing deep learning-based bladder cancer grading model, with accuracy, F1 value, and AUC values of 0.9488, 0.9426, and 0.9459, respectively.

**Conclusion:**

Our proposed SMMF framework combined with MP-MRI information can accurately predict the pathological grading of BCa and can better assist physicians in diagnosing BCa.

## 1 Introduction

Bladder cancer (BCa) is one of the highly prevalent malignant tumors of the urinary system, and its incidence ranks ninth among malignant tumors worldwide (1), among which urothelial cell carcinoma (UCC) is the most common (2). UCC can be divided into high-grade urothelial carcinoma (HGUC) and low-grade urothelial carcinoma (LGUC) (3). HGUC and LGUC have different disease recurrence factors and induce different treatment modalities. For HGUC recurrence, influential factors include lymphovascular infiltration, tumor size, focal prostatic urethral involvement, and variant histology (4). Whereas, Mastroianni, Riccardo et al. demonstrated that gender, multiple tumors, tumor diameter greater than or equal to 3 cm, and European Organization for Research and Treatment of Cancer (EORTC) risk group were significant predictors of recurrence in patients with LGUC (5). The choice of treatment depends largely on the infiltration of the primary tumor, and pathological grading is an important factor in determining the aggressiveness of BCa; most patients with muscle invasive bladder cancer (MIBC) are HGUC, and non-muscle invasive bladder cancer (NMIBC) are usually LGUC (6). The main treatment modality for NMIBC is transurethral resection of bladder tumor (TURBT), combined with bladder perfusion chemotherapy or bladder perfusion immunotherapy depending on the postoperative situation (7). However, the standard treatment for patients with MIBC is radical cystectomy (RC) with pelvic lymph node dissection, supplemented by a variety of therapies including chemotherapy, radiotherapy, immunotherapy, and targeted therapies, depending on the patient’s condition (8). Therefore, accurate assessment of the pathological grading of bladder tumor tissue is important for developing surgical strategies, predicting prognosis, and establishing reasonable follow-up strategies.

Multi-parametric magnetic resonance imaging (MP-MRI) has become a favorable medical diagnostic tool for the study of BCa lesions (9). MP-MRI contains multiple sequences such as T2-weighted imaging (T2WI), diffusion-weighted imaging (DWI), apparent diffusion coefficient, (ADC), and dynamic contrast enhancement (DCE). Each sequence captures specific features related to BCa. To achieve a more comprehensive set of features, researchers have explored the potential correlation between MP-MRI as a means of enhancing the diagnosis of BCa. Currently, MP-MRI has been shown to improve the assessment of BCa for staging (10–12), adjuvant chemotherapy (13), and grading (9, 14, 15). For instance, in a study by Zhang et al. (9), texture features were extracted from DWI and ADC and then combined with a Support Vector Machine (SVM) classifier to assess BCa grading. Similarly, Wang et al. (14) extracted features from T2WI and DWI to create distinct subsets of features, ultimately constructing a joint model that exhibited high accuracy in BCa grading. Xu et al. (15) utilized T2WI, DWI, and ADC to extract features and constructed an optimal discriminant model for determining the degree of muscle infiltration of BCa using the support vector machine with recursive feature elimination (SVM-RFE) algorithm and the synthetic minority oversampling technique (SMOTE). Although the methods described above can be effective in the diagnosis of BCa, they require manual feature extraction and don’t effectively utilize the information contained in MP-MRI.

Convolutional neural networks (CNNs), the conventional deep learning framework, are progressively supplanting earlier machine learning methods as the predominant tools for medical image classification (16). For medical image classification, networks such as deep residual network(ResNet) (17), extreme inception(Xception) (18), and dense convolutional network (DenseNet) (19), which perform well on natural image classification tasks, are commonly used. Nonetheless, medical images encompass multiple organs and intricate image textures, rendering it challenging for CNN networks to swiftly extract relevant information for disease diagnosis. To make CNNs focus on important region information, a series of attention mechanisms have been proposed, such as squeeze-and-excitation networks (SENet) (20), efficient channel attention for deep convolutional neural networks (ECANet) (21), Convolutional Attention Module(CBAM) (22), and so on. Distinct from the above approaches, the self-attention mechanism reduces the dependence on external information and is better at capturing inter-feature correlations. Vaswani et al. (23) first proposed self-attention and applied it to the field of natural language processing (NLP). Borrowing ideas from NLP, self-attention-based vision transformer (VIT) (24) and Swim_transformer (25) have been well applied to image classification tasks. Wang et al. (26) introduced a multi-stage fundus image classification model, which combines CNN and an attention mechanism to enhance the accuracy of fundus disease recognition.

Many deep learning algorithms have been applied to the field of BCa classification. Jansen et al. (27) first used a U-Net segmentation network to detect uroepithelial cells and then used pre-trained visual geometry group (VGG16) to build a classification network to grade UCC. This study demonstrates that the deep learning algorithm can be used for the automated detection and grading of UCC. Since then, more and more scholars have applied deep learning methods to the diagnosis of BCa tumors. To overcome the overfitting problem associated with training on small medical datasets, researchers have used the ImageNet pre-training architecture to distinguish the degree of BCa infiltration and the grading of BCa (28, 29). Garcia et al. (30) introduced a deep clustering framework that employs an unsupervised learning strategy for the hierarchical diagnosis of histopathological images related to BCa, effectively reducing the need for costly data tagging. Zhang et al. (31) pioneered the use of 3D CNN networks for predicting BCa muscle invasiveness. They successfully extracted features using dense blocks and pyramidal structures, capturing both local and global features. In addition, Liu et al. (32) developed an end-to-end ResNet dual-objective prediction model for BCa staging and grading. They applied superresolution and nonlocal attention models to improve the quality of MRI images and enhance the model’s ability to perceive features at greater distances. Nevertheless, the studies on bladder cancer mentioned above are all rooted in single MRI sequences or single-modality data, thus overlooking the potential specific information offered by MP-MRI.

**Figure 1:**
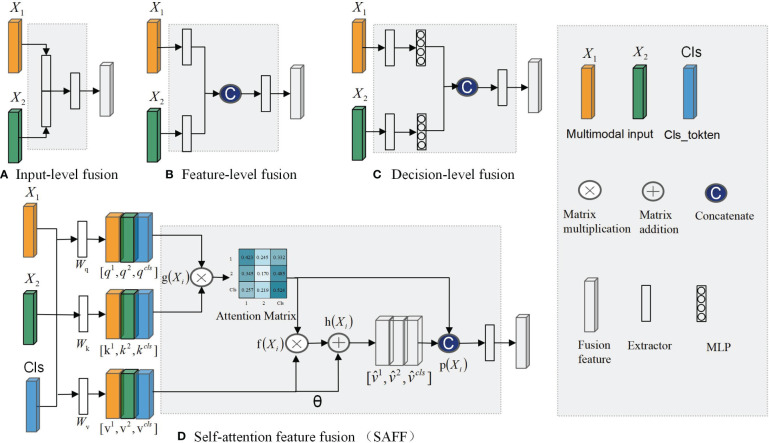
Multimodal feature fusion strategy: **(A)** Input-level fusion; **(B)** feature-level fusion; **(C)** decision-level fusion; **(D)**SAFF.

The fusion of complementary information from multimodal data can lead to more robust predictions (33). Based on the timing of fusion, it can be categorized into three types: input-level fusion, feature-level fusion, and decision-level fusion (34). **Figure 1A** demonstrates the input-level fusion strategy, which integrates various modal images into a single dataset, allowing the neural network to utilize all information from each image and preserve the original features to the fullest extent. The potential of input-level fusion has been shown in several studies to obtain comprehensive features (35, 36). As shown in **Figure 1B**, feature-level fusion is a simpler feature fusion method, which usually uses the “Concate” or “Add” method to stitch together the feature maps extracted from different neural network branches. These studies (37, 38) all fused separately extracted dermoscopic and clinical image features in the final stage of the network. Chen et al. (39) classified a mixture of features extracted from different modal images and confirmed that combining multimodal features led to better results. As shown in **Figure 1C**, decision-level fusion completes classification independently on different pattern data and fuses the recognition results of multiple classifiers to make the global optimal decision. Ilhan et al. (40) used deep features extracted by a single CNN connected into a feature vector, which was then fed into the classifier, and finally, a majority voting pattern was used to combine the decisions of the classifier. Shachor et al. (41) set up a gate network to dynamically combine each decision and make predictions.

Every feature fusion method supplies distinct modalities of specific information for disease diagnosis, yet each one exhibits varying degrees of limitations. Input-level fusion requires a complex data preprocessing process, which increases the difficulty of implementation and debugging. Feature-level fusion usually leads to an increase of feature dimensionality and an increase of computational resource requirements. In decision-level fusion, the outputs of each mode are independent, lacking inter-modal correlations.

To address the aforementioned issue, this study introduces a self-attention-based MP-MRI feature fusion framework (SMMF) for the grading of BCa. In this framework: first, a plug-and-play multiscale attention (MA) model is designed in this study, which is embedded at the end of the neural network in the feature extraction phase to further extract feature information of the expanded T2WI and DCE images; second, a self-attention-based improved feature fusion (SAFF) strategy is proposed, unlike the traditional feature fusion strategy, which not only fuses common and specific features of MP-MRI but also enhances the interaction between features, as shown in **Figure 1D**; Finally, the accurate grading of BCa images is output after two fully connected layers. The main contributions of this study are as follows:

(1) A novel feature fusion framework called SMMF is proposed to fill the gap of MP-MRI in BCa deep learning. The method can fully extract and fuse MP-MRI features, which improves the accuracy of BCa grading.
(2) A plug-and-play MA module is designed, which incorporates InceptionV1 block, CBAM, and hopping connections.MA deepens and widens the network to further extract rich multi-scale features, which enhances the model’s perceptual capability.
(3) A new feature fusion strategy SAFF is constructed, which can effectively fuse the common features and complementary features of MP-MRI and enhance the interactivity of feature fusion.

## 2 Methods

**Figure 2:**
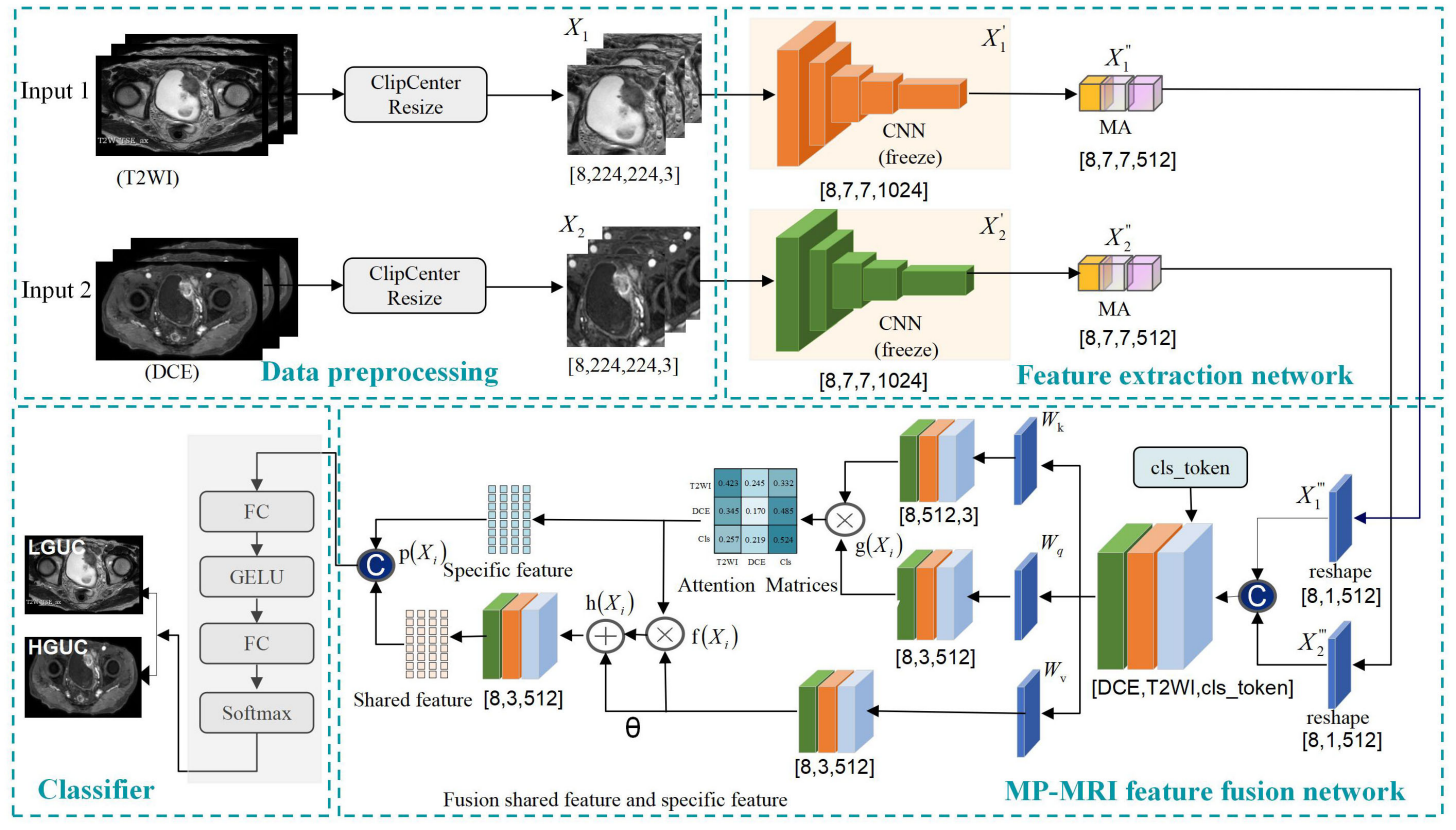
Self-attention-based MP-MRI feature fusion (SMMF) framework. CNN: extraction of underlying BCa features; multi-scale attention model (MA): extraction of rich multi-scale features; Self-attention feature fusion model (SAFF): fusion of MP-MRI features.

To effectively fuse MP-MRI features, this study proposes a self-attention-based MP-MRI feature fusion framework (SMMF), as shown in **Figure 2**. The architecture includes four parts: data preprocessing, feature extraction network, feature fusion, and classifier. First, to make the input data more adaptable to the model, center cropping and data expansion are used to enhance the image quality. Then, two independent feature extractors are used to extract image features from different sequences respectively, based on which a multi-scale attention (MA) module is designed to further extract high-level semantic features. Subsequently, to better capture and fuse the common and complementary features of MP-MRI, the self-attention feature fusion (SAFF) strategy is designed. Finally, a two-layer classifier is used to output the classification results of LGUC and HGUC. The details of the methodology are given below.

### 2.1 Self-attention-based MP-MRI feature fusion framework

#### 2.1.1 Data preprocessing

In order to make the input data more adaptable to the model, preprocessing operations are performed on the data. Center cropping and data expansion (random vertical flip, random horizontal flip, luminance adjustment, and miscut transformation) are used to enhance the image quality, the input image is $X_{1},X_{2}\in R^{B\times H\times W\times C}$ , where $X_{1}$ denotes T2WI, $X_{2}$ denotes DCE, *B* is batchsize, *H* is the height of the image, *W* is the width of the image, and *C* is the number of channels, and the size of the input image is united as [*B=*8, *H*=224, *W*=224, *C*= 3].

#### 2.1.2 Feature extraction

In the feature extraction part, two independent branching networks are first used to extract feature information for T2WI and DCE respectively, and a feature map of size [8,7,7,1024] is obtained. Then, to further enhance the feature extraction capability of the network, an MA module is designed at the end of the network model, which is described in detail in Section 2.2 of this study.

#### 2.1.3 Feature fusion

Since the self-attention module can only accept two-dimensional feature vectors, the extracted T2WI and DCE features must undergo a reshape operation before they can be spliced and input into the self-attention module. The T2WI and DCE features are compressed to [8,1,512], respectively, and are spliced with the class coding vectors (cls_token) to obtain the multimodal feature vectors with the size of [8,3,512]. The three-parameter matrix of self-attention, and the obtained attention matrix are utilized to adjust the correlation coefficients among modal features to better capture and fuse the common and specific features of MP-MRI, and this module is described in detail in Section 2.3 of this study.

#### 2.1.4 Classifier

To mitigate gradient descent, two fully connected layers are used to output classification results for LGUC and HGUC.

### 2.2 Multiscale attention module

**Figure 3:**
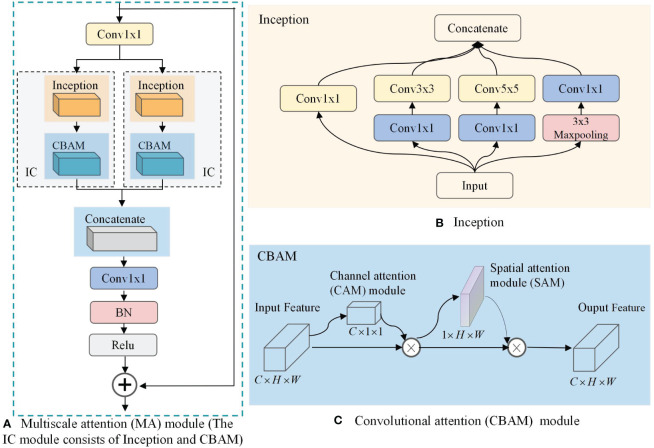
Multiscale attention (MA) module. **(A)** MA; **(B)** Inception; **(C)** Convolutional Attention Module(CBAM).

Increasing the number of layers of CNN can improve the model performance, but if the number of layers is too deep, it will lead to parameter explosion and overfitting, which will reduce the final performance. To further extract rich high-level features, a multi-scale attention MA block incorporating InceptionV1 block, CBAM attention mechanism, and jump connection techniques is proposed. Its structure is shown in **Figure 3A**.

As can be seen in **Figure 3A**, the MA block is composed of two 1×1 convolutions, a jump connection, and two IC blocks. The 1×1 convolution is used to reduce the number of output channels of the CNN from 1024 to 512 to reduce the computational effort of the IC block. The jump connection enables image feature reuse for combining features in different paths, connecting low-level features with high-level features, and helping the network to obtain comprehensive features. In addition, it can effectively mitigate gradient disappearance, enhance feature transfer and reuse, and reduce the number of parameters to avoid possible information blocking in the residual structure. We use a parallel structure in the MA block to combine two IC models, which are newly constructed functional models used to extract multi-scale information from BCa images in this study, by combining the InceptionV1 block and the CBAM attention mechanism. As shown in **Figure 3B**, the InceptionV1 block incorporates three different sizes of convolutional kernels, namely 1×1 , 3×3 , and 5×5 , within the same layer of the network. This enables the block to capture features of varying sizes and enhances the model’s ability to perceive information of different scales. On this basis, the introduction of CBAM can change the way resources are allocated so that features with greater contribution can be extracted. As shown in **Figure 3C**, through the combination of the channel attention (CAM) model and the spatial attention (SAM) model, CBAM can consider both channel information and spatial information of the feature map to extract more accurate and distinguishable features.

### 2.3 Self-attention feature fusion strategy

The core element of the self-attention structure is the self-attention mechanism for establishing relationships between data nodes (42). Regardless of the heterogeneity between nodes, the relationship between them can be established by mapping them into feature vectors. Therefore, MP-MRI can utilize the self-attention structure to compute the correlation between the features of different MRI sequences, responding to the common and specific features among different sequences. As shown in **Figure 1D**, we propose a self-attentive feature fusion strategy (SAFF) to enhance the interaction between features by fusing commonality and specificity features among MP-MRIs. SAFF serves as a bridge connecting the T2WI and DCE features to achieve mutual compensation of the information and improve classification accuracy.

First, the combination of CNN and self-attention is achieved through a Reshape operation. A feature map of size [8,7,7,512] is converted to a feature vector containing all information [8,512] by global average pooling and the dimensionality is expanded to [8,1,512] for input SAFF.

Second, the relevant attention scores for DCE and T2WI are computed using self-attention, which provides an inter-modal specific feature representation. Where $W_{q}$ , $W_{k}$ , and $W_{v}$ are the parameter matrices used to generate queries, keys, and values, respectively, which are updated by backpropagation of the network during model training. The DCE and T2WI and the corresponding class codes are spliced to obtain fused MP-MRI features with dimensions [8,3,512]. The MP-MRI features are input into Equation 1 to compute the attention matrix of *Q* and *K* between different sequences to obtain the attention coefficient $g\left(X_{i}\right)$ between different sequences, and then input into the *Softmax()* function to normalize the specificity feature values.


(1) $$g\left(X_{i}\right)=Softmax\left(\frac{Q_{X_{i}}K_{X_{i}}^{T}}{\sqrt{d}}\right)$$

Then, to extract common features from different MRI sequences, it is necessary to aggregate the MP-MRI feature vectors. Multiplying the attention matrix with V results in common features $f\left(X_{i}\right)$ for various sequences via Equation 2. Building upon this, the study further optimizes the common features by incorporating some of the original features using a short connection method. Here, θ represents the coefficient for preserving the relevance of the original features, and multiplying θ with V calculates the importance of the original features. This transformation converts the common features from $f\left(X_{i}\right)$ to $h\left(X_{i}\right)$ expression via Equation 3.


(2) $$f\left(X_{i}\right)=Softmax\left(\frac{Q_{X_{i}}K_{X_{i}}^{T}}{\sqrt{d}}\right)V_{X_{i}}$$

(3) $$h\left(X_{i}\right)=V_{X_{i}}*\theta +f\left(X_{i}\right)$$

Finally, the specific features and the common features are spliced to complete the feature fusion of the two MRI sequences via Equation 4.


(4) $$p\left(X_{i}\right)=g\left(X_{i}\right)+h\left(X_{i}\right)$$

SAFF dynamically focuses on the key features of MP-MRI and adaptively adjusts the correlation coefficients to better fuse specific features and common features to fuse different MP-MRI sequence features.

## 3 Experimental results

### 3.1 Dataset

#### 3.1.1 Patient selection

BCa patients admitted to the Second Affiliated Hospital of Kunming Medical University from April 2019 to February 2021 were collected retrospectively. Studies involving human subjects were reviewed and approved by the Ethics Committee and written informed consent requirements for participation were waived by the Ethics Committee. The ethics committee waived the requirement of written informed consent for participation. Inclusion criteria: (1) preoperative MRI examination with good image quality; (2) no history of BCa treatment before the study; (3) postoperative pathological confirmation of UCC (HGUC or LGUC); and (4) lesion diameter >0.5 cm to ensure accurate manual outlining of the volume of interest (VOI). Exclusion criteria: (1) patients with no or incomplete pathological data and pathologically confirmed non-urothelial cell carcinoma; (2) patients who did not undergo preoperative MRI or who underwent MRI more than 2 weeks before surgery; (3) Any therapeutic intervention for tumor progressions such as chemotherapy, radiotherapy, or bacillus calmette–guérin (BCG) vaccine before treatment; (4) Lesions<0.5 cm in diameter or prostrate growth making it difficult to outline. After screening, the dataset consisted of 138 bladder patients, the majority of whom were male (76.1%); the mean age was 65 years (age range 30-86). Of these patients, 76 (55.1%) had a tumor pathology grade of HGUC and 62 (44.9%) had a tumor grade of LGUC. Among these patients, single tumors were more or less 82 (59.4%) and multiple tumors were more or less 56 (40.6%). The demographic and histopathological characteristics of the included patients are shown in **Table 1**.

**Table 1:** Demographic and histopathological characteristics of included patients.

Characteristics (n=138)	Median (Min-Max)/n (%)
Age(years)	65 (30-86)
Gender	0.8782
Male	105 (76.1)
Female	33 (23.9)
Grading	
HGUC	76 (55.1)
LGUC	62 (44.9)
Number of tumors	
Single	82 (59.4)
Multiple	56 (40.6)

#### 3.1.2 MRI acquisition protocol

**Figure 4:**
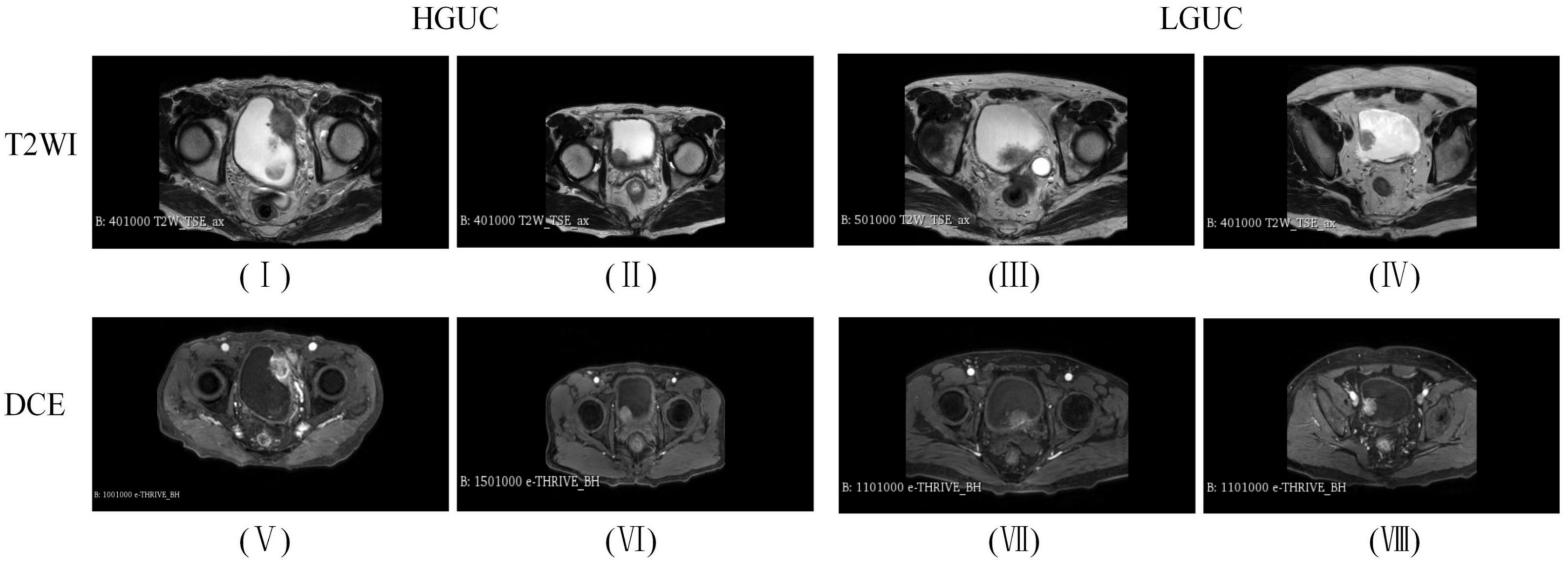
Images of BCa sample data.

All BCa patients underwent MP-MRI using a 3.0 T MRI scanner before biopsy, including DICOM images of T2WI and the DCE arterial phase (images acquired within 40-60 s after contrast injection). As shown in **Figure 4**, (I) (II) is the HGUC image of the T2WI sequence; (III) (IV) is the LGUC image of the T2WI sequence; (V) (VI) is the HGUC image of the DCE sequence; (VII) (VIII) is the LGUC image of DCE sequence. T2WI can illustrate detailed structural information of the lesion and bladder wall. The detrusor muscle of bladder become banded with low signal, thus showing the general outline of the bladder. the importance of DCE in assessing tumor aggressiveness depends on the neovascularization of the tumor. The more neovascularization, the higher the tumor stage and grade.

#### 3.1.3 Data preprocessing

The abdominal BCa data (DICOM format) of each patient were imported into 3D Slicer (version 4.11.20210226) software. This study was based on MR-MRI (T2WI and DCE), and the slice images in which the physician outlined the region of interest (ROI) were selected to constitute the dataset. The number of thick T2WI slices ranged from 15-24 (number of slices with ROI 1-3); the number of DCE slices ranged from 65-70 (number of slices with ROI 1-5). The dataset included 76 patients with HGUC (193 slices) and 62 patients with LGUC (123 slices), totaling 316 slices. Since there were also a large number of unrelated organs in the abdominal BCa images, we performed a preprocessing operation of center cropping and standardizing the image size to 224 x 224. The dataset was divided into a train set, validation set, and test set according to the ratio of 8:1:1, and subsequently, the same data augmentation methods were used for them respectively, and the detailed distribution of the data is shown in **Table 2**. 110 cases of the training set (60 cases of HGUC, 50 cases of LGUC), 14 cases of the validation set and (8 cases of HGUC, 6 cases of LGUC) test set of 14 cases (8 cases of HGUC, 6 cases of LGUC). The training set contained slice images (155 HGUCs expanded to 743 and 99 LGUCs expanded to 665), the validation set contained slice images (19 HGUCs expanded to 94 and 12 LGUCs expanded to 82), and the test set contained slice images (19 HGUCs expanded to 94 and 12 LGUCs expanded to 82).

**Table 2:** The data distribution.

	Train set(n=110)		Validation set(n=14)		Test set(n=14)		
	HGUC (n=60)	LGUC (n=50)	HGUC (n=8)	LGUC (n=6)	HGUC (n=8)	LGUC (n=6)	Total (n=138)
Original	155	99	19	12	19	12	316
Augmented	743	665	94	82	94	82	1760

### 3.2 Hyperparameter optimization

The implementation of this experiment is based on the TensorFlow framework (version 2.4.0). Computer configuration parameters: 64-bit OS Windows 10, 32GB running memory, Core i9-11900K processor, NVIDIA RTX 3060 GPU, 12GB video memory, python 3.6.

The algorithm in this study uses an SGD optimizer to update the parameters with a learning rate of 0.01, epochs set to 50, batch size set to 8, and a loss function of cross-entropy loss, L1 regularization and early stopping training.

### 3.3 Choice of backbone model

To select the optimal backbone model, this study compared widely used classification networks, including ResNet (17), Xception (18), DenseNet121 (19), VIT (24), and Swin Transformer (25). MP-MRI (DCE or T2WI) was input into the networks, and the results are presented in **Table 3**. The experimental loss functions, optimizers, and parameter settings were kept consistent.

**Table 3:** Comparison of different classification models.

Model	Dataset	Accuracy	F1	AUC
ResNet50	DCE	0.8352	0.8129	0.8310
	T2WI	0.8806	0.8679	0.8782
Xception	DCE	0.8806	0.8662	0.8774
	T2WI	0.9090	0.9024	0.9087
DenseNet121	DCE	**0.8977**	**0.8941**	**0.8996**
	T2WI	**0.9147**	**0.9079**	**0.9140**
VIT	DCE	0.5739	0.6695	0.5964
	T2WI	0.5682	0.3666	0.5490
Swin_transformer	DCE	0.6648	0.6427	0.6636
	T2WI	0.7045	0.6579	0.6985

The bold values represent the best results of the experiment.

**Figure 5:**
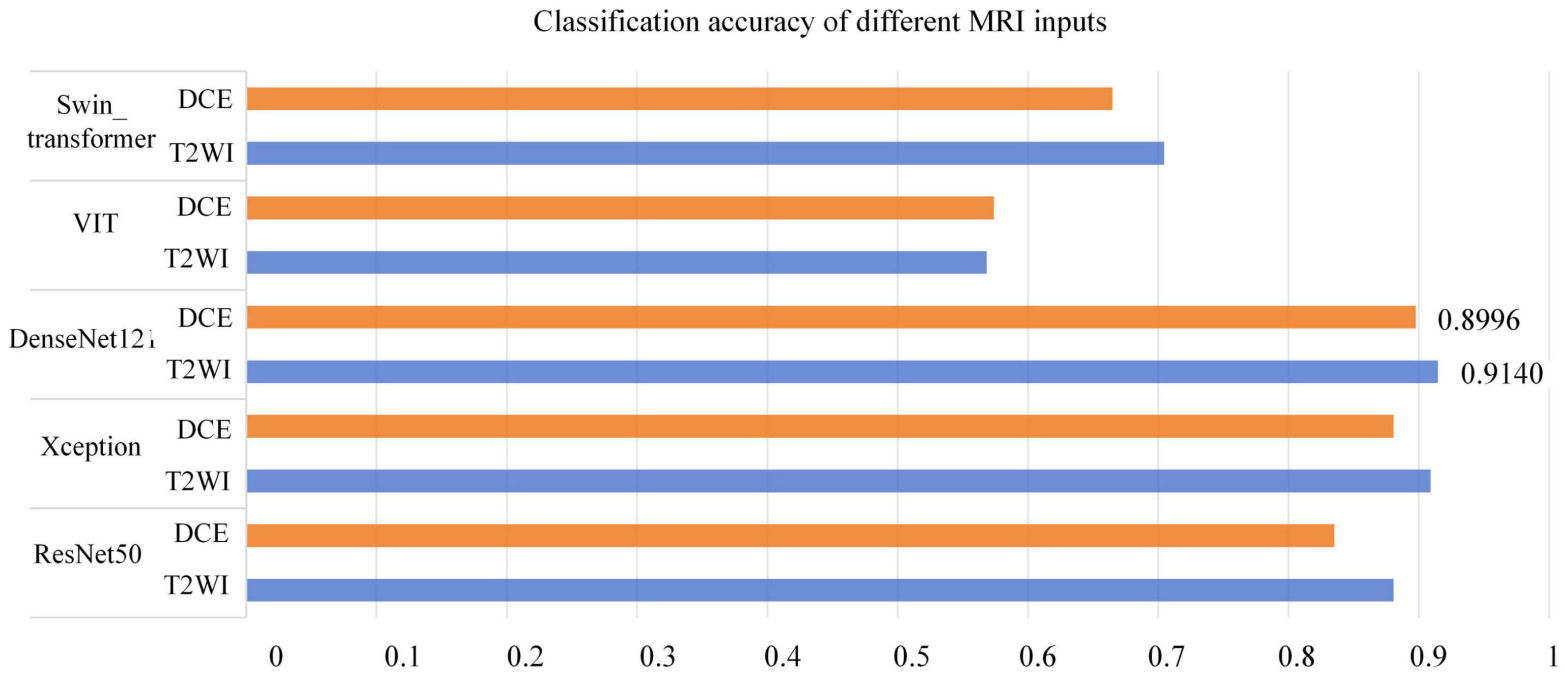
Classification Accuracy of Different MRI Inputs on Multiple Networks (Orange in T2WI, Blue in DCE).

As depicted in **Table 3**, both the T2WI and DCE sequences demonstrated superior classification performance on the DenseNet121 network. When utilizing T2WI as input, the model achieved an Accuracy of 0.9147, F1 of 0.9073, and AUC of 0.9140. When DCE was used as input, the model yielded an Accuracy of 0.8977, F1 of 0.8941, and AUC of 0.8996. **Figure 5** presents a histogram visualization of the classification accuracy across the five models, conspicuously showcasing the consistent superiority of the DenseNet121 model. These findings affirm that the DenseNet121 network is the most suitable choice for BCa image classification. Consequently, we have adopted this network as the primary backbone model for initial feature extraction in our framework.

### 3.4 Comparison of different MRI inputs

To validate the effectiveness of the SMMF framework proposed in this study, different MRI inputs were fed into the network framework, and the results are presented in **Table 4**.

**Table 4:** Comparison of different MRI inputs.

Dataset	Accuracy	F1	AUC
DCE	0.9034	0.8957	0.9026
T2WI	0.9204	0.9125	0.9185
T2WI+DCE	**0.9488**	**0.9426**	**0.9459**

The bold values represent the best results of the experiment.

**Figure 6:**
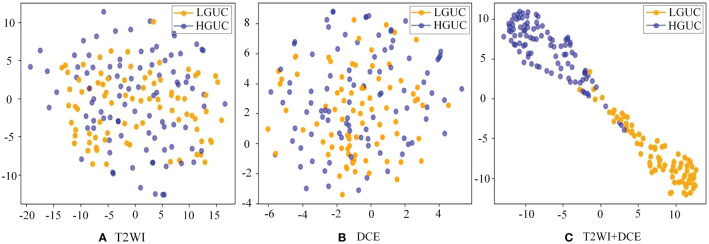
Separability of two MRI sequences data **(A)** original test set separability of T2WI sequences; **(B)** original test set separability of DCE sequences; **(C)** test set separability after SMMF framework classification; blue dots represent HGUC and orange dots represent LGUC.

As demonstrated in **Table 4**, the SMMF framework achieved optimal performance across all classification evaluation metrics, with an Accuracy of 0.9488, F1 of 0.9426, and AUC of 0.9459. The integration of MP-MRI features through the SMMF framework outperformed individual MRI sequence inputs, emphasizing the effectiveness of the SMMF framework for feature fusion. Furthermore, this study employed t-distributed stochastic neighbor embedding (t-SNE) for visual validation of the SMMF framework’s efficacy. As shown in **Figure 6**, this method intuitively reveals that the SMMF framework exhibits high cohesion for HGUC and LGUC, enhancing the separability of the test data.

### 3.5 10-fold cross verification

To better demonstrate the stability and generalization of the proposed model in this study, we conducted experiments using 10-fold cross-validation on a small medical dataset. The data is divided into 10 subsets, and one separate subset is used as the test data of the model, and the corresponding evaluation index is calculated. Of the remaining 9 subsets, 8 subsets are used for training and the remaining subset is used for validation. Cross-validation was repeated 10 times, and each subset was tested once. Detailed results were shown in **Table 4**. As shown in **Table 5**, the variance (Var) of Accuracy, F1 and AUC indicators are verified through 10-fold cross-validation, and they are all relatively small as 0.0001, 0.0002 and 0.0002 respectively. Therefore, the model has better stability and generalization.

**Table 5:** The result of 10-fold cross-validation.

Fold	Accuracy	F1	AUC	
1	0.9545	0.9518	0.9544	
2	0.9375	0.9356	0.9389	
3	0.9261	0.9182	0.9236	
4	0.9432	0.9405	0.9436	
5	0.9318	0.9250	0.9297	
6	0.9261	0.9231	0.9269	
7	0.9375	0.9378	0.9409	
8	0.9375	0.9333	0.9370	
9	0.9602	0.9570	0.9591	
10	0.9204	0.9102	0.9170	
Mean	0.9375	0.9332	0.9371	
Var	**0.0001**	**0.0002**	**0.0002**	

The bold values represent the best results of the experiment.

### 3.6 Comparison of different feature fusion strategies

**Figure 7:**
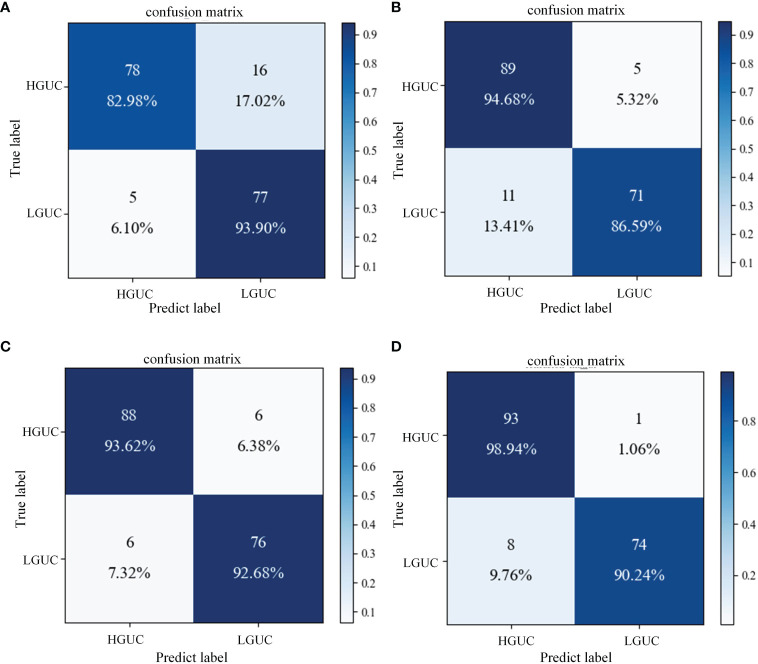
Confusion matrix of different feature fusion strategies. **(A)** Input-level fusion; **(B)** Feature-level fusion; **(C)** Decision-level fusion; **(D)** SAFF.

The proposed SAFF model is compared with the three most frequently used feature fusion strategies in **Figure 1** (**Figure 1A** input-level fusion, **Figure 1B** feature-level fusion, and **Figure 1C** decision-level fusion). **Table 2** shows the results of the comparison of different feature fusion strategies. The confusion matrices for the four feature fusion strategies are shown in **Figures 7A–D**, and the specific classification results of HGUC and LGUC can be seen by the following confusion matrices.

As seen in **Table 6**, the input-level fusion strategy has the lowest classification accuracy at 0.8806. decision-level fusion can obtain better results by weighting different modal branching networks, and the test accuracy reaches 0.9318, which is second only to SAFF. all evaluation indexes of SAFF are better than the above three strategies, and the classification accuracy, F1 value, and AUC are 0.9488, 0.9426, and 0.9459, respectively. Through the comparison of the above experiments, it is further verified that SAFF has a stronger capability of feature fusion.

**Table 6:** Comparison results of feature fusion strategies.

Model	Accuracy	F1	AUC
Input-level fusion	0.8806	0.8799	0.8844
Feature-level fusion	0.9090	0.8987	0.9063
Decision-level fusion	0.9318	0.9268	0.9360
**SAFF**	**0.9488**	**0.9426**	**0.9459**

The bold values represent the best results of the experiment.

### 3.7 Ablation experiments

**Figure 8:**
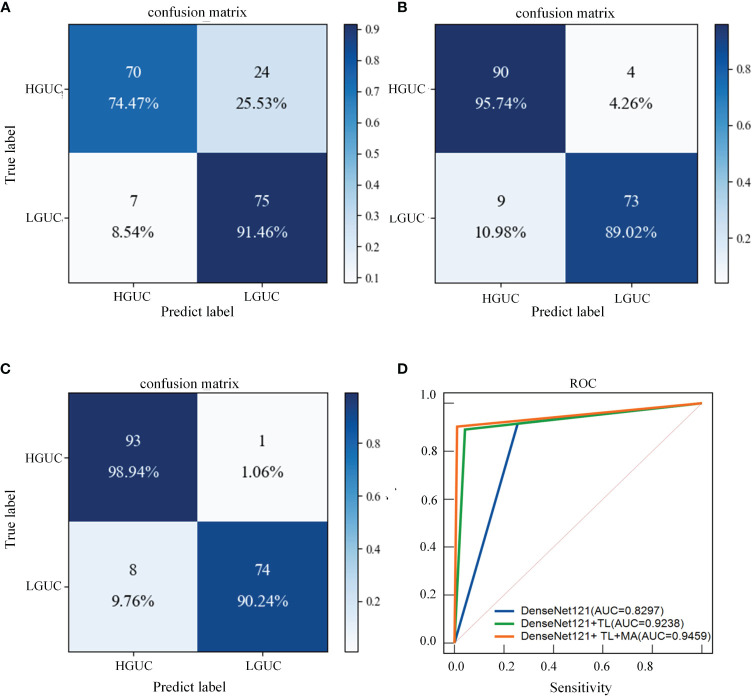
Confusion matrix and ROC curves for different deep learning methods. **(A)** DenseNet121; **(B)** DenseNet121+ TL; **(C)** DenseNet121+ TL + MA. **(D)** Blue line: DenseNet121; green line: DenseNet121+ TL; orange line: DenseNet121+ TL+ MA.

As shown in **Table 7**, the following experiments were conducted to validate the performance of Transfer Learning (TL) and the MA block we designed. (I): Exclude TL, and use DenseNet121 as the baseline model. (DenseNet121); (II): Introduce TL based on Experiment I. (DenseNet121+ TL); (III): MA block is added to the use of TL (DenseNet121+ TL+ MA). The following experiments are all implemented on the MP-MRI (T2WI+DCE) dataset, and the experimental parameters are set the same as the above experiments. Additionally, the confusion matrices of three different deep learning methods are shown in **Figures 8A–C**, and the ROC curves of the three methods are shown in **Figure 8D** to show the classification performance. As can be seen in **Table 6**, training with the TL method results in better classification performance with an accuracy improvement of about 10%, which illustrates the effectiveness of the TL approach. By training the model using the ImageNet dataset, the model is given a suitable initialization parameter, which speeds up the convergence of the model and improves the accuracy of the model classification. Secondly, based on the use of the TL method, the MA block is introduced and the average accuracy is improved by 2.27% on the DenseNet121 model, which proves that the algorithm proposed in this study can better extract BCa features and improve the classification accuracy. From **Figure 8C**, we can see that the introduction of the MA block has better recognition ability for both positive and negative samples, which is better than the first two methods.

**Table 7:** Comparison of different deep learning methods.

Model	Accuracy	F1	AUC
DenseNet121	0.8238	0.8287	0.8297
DenseNet121+ TL	0.9261	0.9182	0.9238
**DenseNet121+ TL+ MA(Ours)**	**0.9488**	**0.9426**	**0.9459**

The bold values represent the best results of the experiment.

### 3.8 Comparison with related work

To demonstrate the superiority of the proposed MP-MRI feature fusion framework in BCa diagnosis, we compared the results for the same diagnostic task with other methods (28, 32). The results in **Table 8** demonstrate that our framework outperformed the others in all evaluation metrics for BCa grading diagnosis. This further substantiates the advantage of the proposed SMMF framework in BCa diagnosis.

**Table 8:** Comparison with related research methods.

Method	T2WI			DCE		
	Accuracy	F1	AUC	Accuracy	F1	AUC
Ali et al. (2021) (28)	0.8806	0.8679	0.8782	0.8352	0.8129	0.8310
Liu et al. (2022) (32)	0.8864	0.8824	0.8882	0.8920	0.8790	0.8888
Ours	**0.9488**	**0.9426**	**0.9459**	**0.9488**	**0.9426**	**0.9459**

The bold values represent the best results of the experiment.

## 4 Discussion

To be able to effectively utilize MP-MRI features for BCa grading, this study proposes a self-attention-based MP-MRI feature fusion framework (SMMF). We conducted experiments on the MP-MRI dataset of BCa and discussed the results based on the above experiments.

As shown in **Table 3**, this study conducted experiments on three classic CNN networks and two Transformer networks with broader receptive fields for comparison. The results indicate that the DenseNet121 network exhibited the best performance, while the classification performance on the Transformer networks was relatively poorer. This discrepancy can be attributed to the fact that, in contrast to other CNN networks, DenseNet121 employs a more aggressive dense connectivity mechanism, establishing dense connections between all layers in the CNN, facilitating feature reuse through channel-wise connections, and maximizing the utilization of information contained in both shallow and deep feature maps, thereby achieving superior classification performance. However, when compared to CNN networks, Transformer networks, while capable of learning global information more effectively, often require larger training datasets, which is why their performance tends to be less favorable on small-sample datasets such as medical datasets.

Furthermore, we validated the proposed SMMF framework under different MRI inputs. The results in **Table 4** demonstrate that simultaneous input of T2WI and DCE sequence data into the SMMF framework outperforms single-sequence input. The reasons for this improved performance are as follows: Firstly, different MRI sequence images exhibit varying sensitivities to BCa and can provide complementary information. T2WI can offer detailed information about lesion areas and bladder wall structures, while DCE can depict tumor staging and grading by illustrating the degree of tumor angiogenesis (43). Secondly, our proposed SMMF framework, based on robust feature extraction networks and feature fusion strategies, effectively extracts and fuses common and complementary features between MP-MRI, thus enhancing the model’s classification performance. To further validate the stability and generalization of the model on the small medical dataset, we conducted 10-fold cross-validation for verification. The experimental results in **Table 5** demonstrate that the model not only exhibits good classification performance but also demonstrates strong stability and generalization.

Additionally, this study further compared three widely used feature fusion strategies in existing research, namely input-level fusion, feature-level fusion, and decision-level fusion. As shown in **Table 6**, the proposed method achieves competitive results. The input-level fusion strategy has the lowest classification accuracy of 0.8806. The method fuses different MRI sequences into one dataset, which expands the sample size but the output feature maps contain a large amount of redundant information. At the same time, the method did not pay sufficient attention to the contributing large features, which led to poor classification results. Feature-level fusion is performed for DCE and T2WI respectively, and the features are directly stitched together using the “Concate” operation. However, the feature extraction process of the two MRI sequences is independent and lacks interactivity, which leads to limited characterization capability of feature extraction. Decision-level fusion obtained better results by weighting the branching network of different MRI sequences, but the outputs of decision-level fusion were independent of each other, ignoring the complex correlation between different modalities. Different from the above three feature fusion strategies, SAFF first rightly focuses on two sequence image features, T2WI and DCE, through a self-attentive mechanism, and then interactively fuses the common and specific features of both to obtain the best classification performance on BCa data.

The better classification performance achieved in this study cannot be achieved without the contribution of the MA block, which makes up for the shortcomings of the CNN and further deepens and broadens the neural network. As can be seen from **Table 7**, although DenseNet121 can achieve a certain classification accuracy, it cannot increase the number of convolutional layers much due to the limitation of the video memory of the device. However, the MA block extracts higher-level multiscale features through the Inception model, which enriches the features extracted by the network. In addition, the CBAM model connects the channel attention and spatial attention mechanisms, enabling the network to focus on features and spatial locations, thus improving the model’s accuracy.

Finally, in **Table 8**, we compared the method proposed in this study with the existing deep learning-based BCa grading method (28, 32). All of the aforementioned methods conducted experiments using single-sequence MRI or single-modality data. Due to the inconsistency of the datasets, such comparisons are unfair. Nevertheless, the MP-MRI fusion approach consistently achieved the best classification results, offering valuable insights for future research in the domain of bladder cancer grading and diagnosis. This underscores the significance of MP-MRI as a pivotal avenue for future studies in bladder cancer grading and diagnosis.

There are some limitations in this study: first, the sample size in this study is relatively small and the amount of data used for training on the BCa dataset is limited. Second, the SMMF framework proposed in this study was specifically designed for the BCa dataset and was not validated on other datasets. Third, only T2WI and DCE data were used in this study, and other combinations of MRI sequences were not explored, and it cannot be concluded that the combination of T2WI and DCE is the optimal combination.

## 5 Conclusions

This study proposes a self-attention-based MP-MRI fusion framework (SMMF) that integrates DCE and T2WI image features for the classification of BCa. The framework mainly consists of a feature extraction network and a feature fusion network. The former further extracts richer multi-scale features by embedding the MA block at the end of the network; the latter uses self-attention to adjust the proportion of different sequences in the feature fusion, thus improving the performance of the model. The experimental results show that the SMMF framework can effectively extract and fuse T2WI and DCE features by combining data enhancement, MA block, and SAFF model, and the classification accuracy, F1 score, and AUC are 0.9488, 0.9426, and 0.9459, respectively, which are better than other comparative CNN classification models. SMMF framework can provide more comprehensive information to achieve accurate prediction of BCa pathological grading and better assist physicians in BCa diagnosis.

## Data availability statement

The data analyzed in this study is subject to the following licenses/restrictions: The datasets generated during this study are not publicly available due to privacy and ethical considerations; however, anonymized data can be provided by the corresponding author upon reasonable request and with the approval of the ethics committee. Researchers interested in accessing the data should contact the corresponding author for further information. Requests to access these datasets should be directed to jfenghe@foxmail.com.

## Ethics statement

Written informed consent was obtained from the individual(s) for the publication of any potentially identifiable images or data included in this article.

## Author contributions

TT: Writing – original draft, Validation, Software, Methodology, Investigation, Formal analysis, Data curation. YC: Writing – review & editing, Data curation. YS: Writing – review & editing, Data curation. JFH: Writing – review & editing, Validation, Supervision, Funding acquisition. JGH: Writing – review & editing, Validation, Supervision.
