# Supplementary material for: SMMF: a self-attention-based multi-parametric MRI feature fusion framework for the diagnosis of bladder cancer grading
Source: Front Oncol. 2024 Mar 7;14:1337186. doi: 10.3389/fonc.2024.1337186 (PMC10955083; doi:10.3389/fonc.2024.1337186)
Supplement: Supplementary file 1 [file DataSheet_1.docx]

Supplementary Material

# Supplementary Data

Supplementary Material should be uploaded separately on submission. Please include any supplementary data, figures and/or tables.

Supplementary material is not typeset so please ensure that all information is clearly presented, the appropriate caption is included in the file and not in the manuscript, and that the style conforms to the rest of the article.

# Supplementary Figures and Tables

For more information on Supplementary Material and for details on the different file types accepted, please see [here](https://www.frontiersin.org/guidelines/author-guidelines#supplementary-material).

## Supplementary Figures


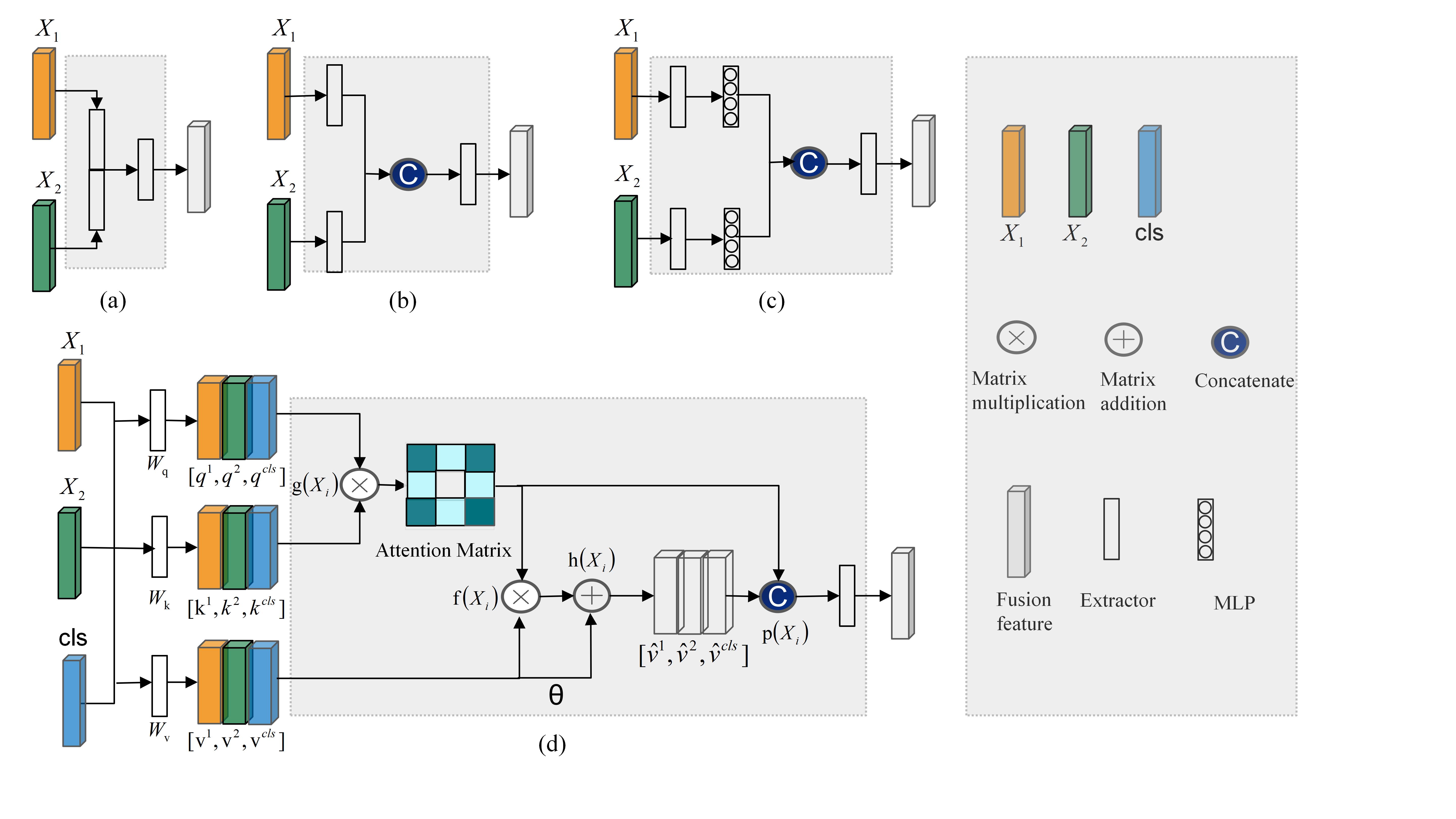


**Supplementary Figure 1.** Multimodal feature fusion strategy:(a) Input-level fusion; (b) feature-level fusion; (c) decision-level fusion; (d)SAFF.


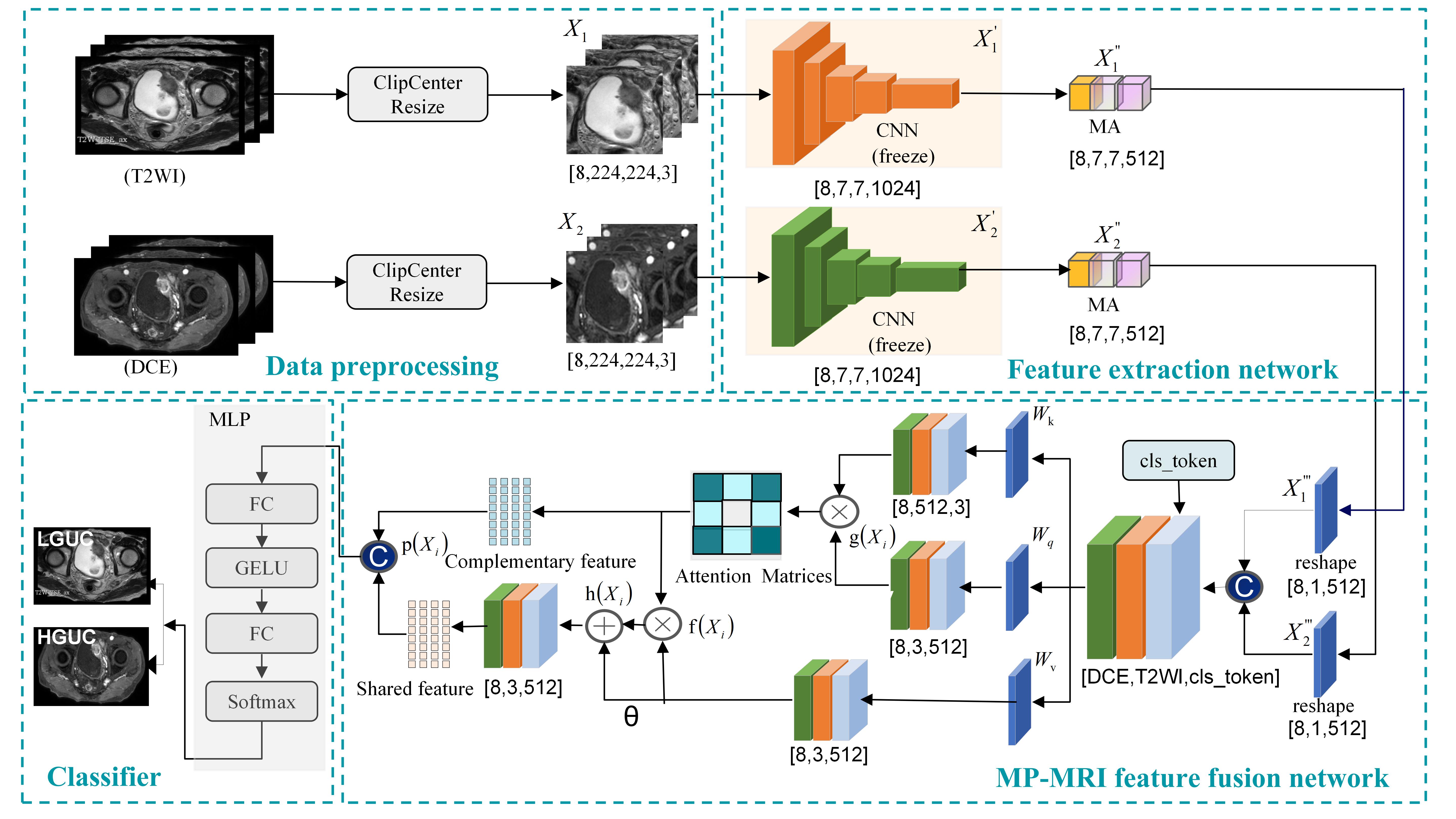


**Supplementary Figure 2.** SMMF framework. CNN: extraction of underlying BCa features; multi-scale attention model (MA): extraction of rich multi-scale features; self-attention feature fusion model (SAFF): fusion of MP-MRI features.


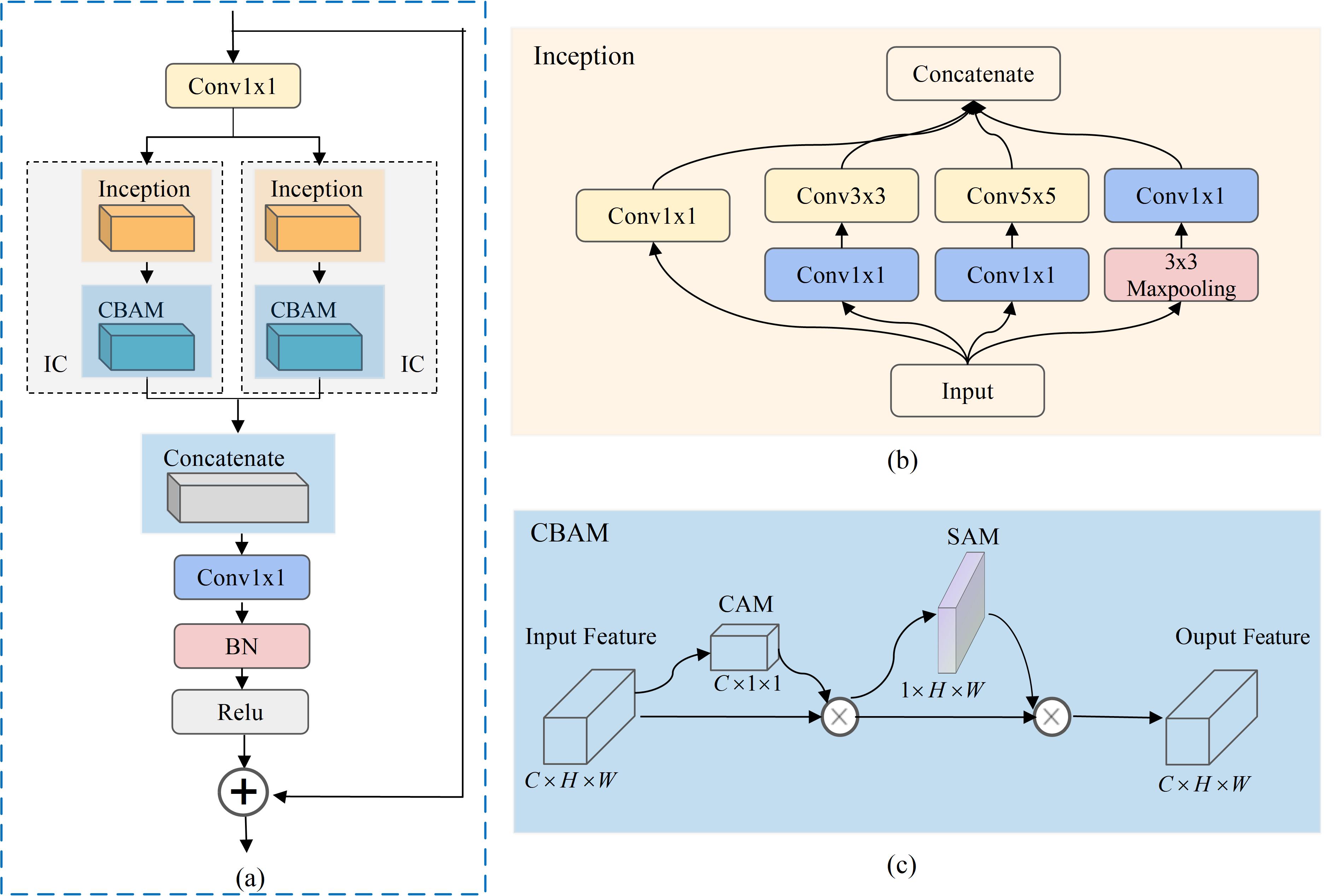


**Supplementary Figure 3.** MA block structure. (a) MA; (b) Inception; (c) CBAM.


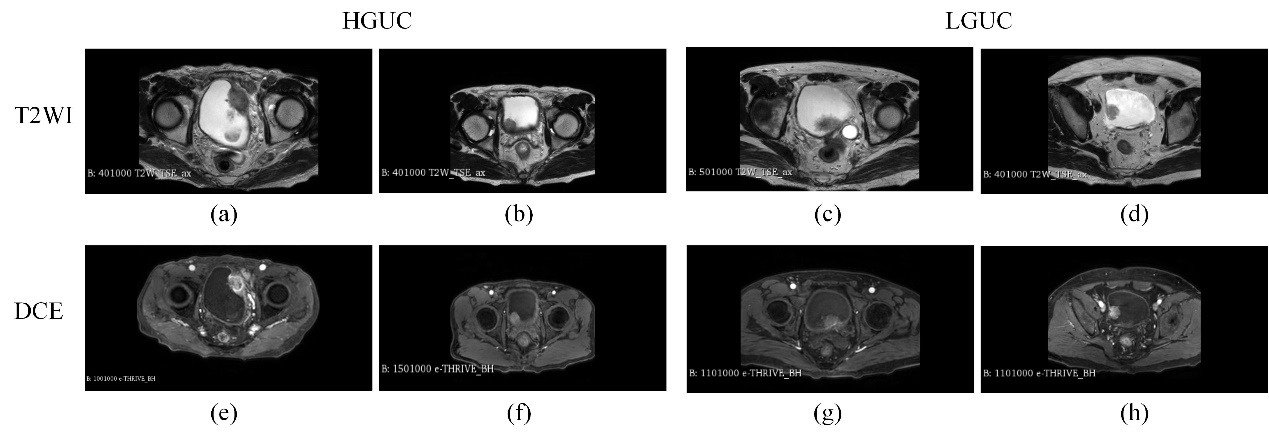


**Supplementary Figure 4.** Images of BCa sample data.


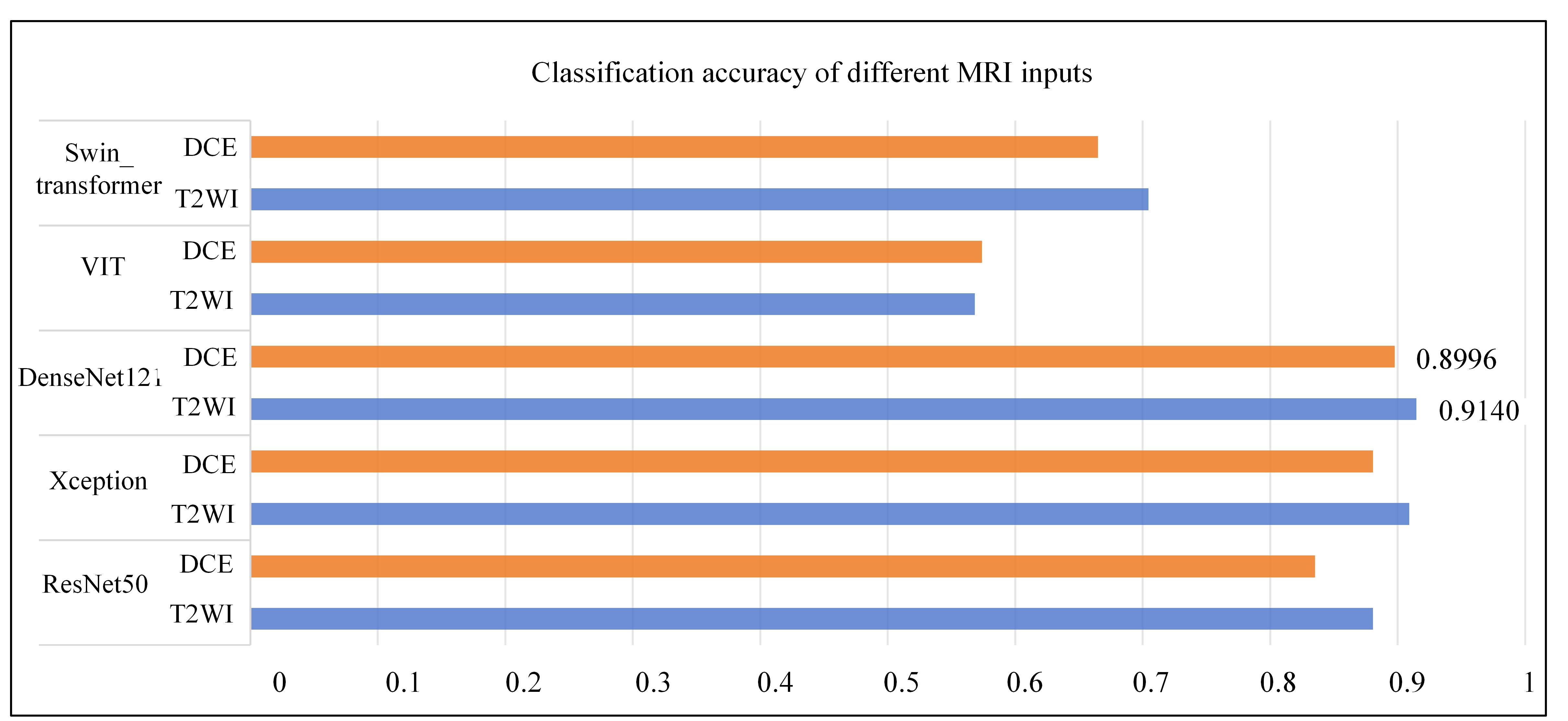


**Supplementary Figure 5.** Classification Accuracy of Different MRI Inputs on Multiple Networks (Orange in T2WI, Blue in DCE).


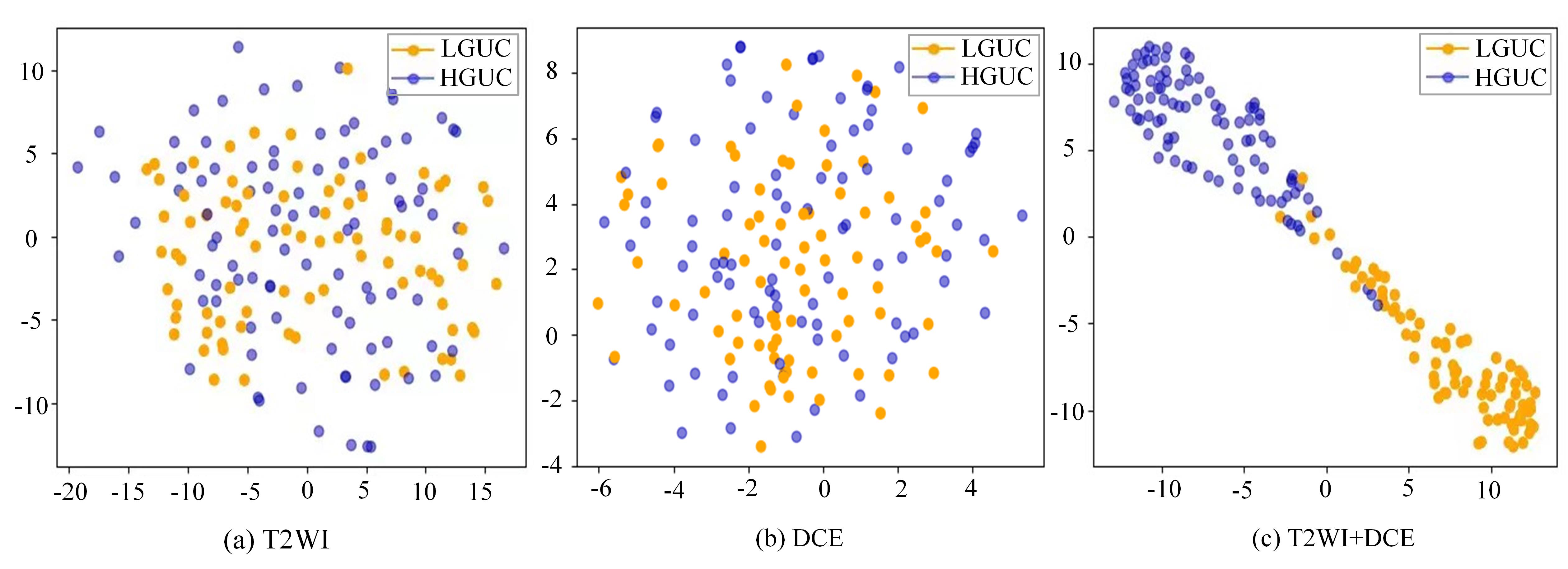


**Supplementary Figure 6.** Separability of two MRI sequences data (a) original test set separability of T2WI sequences; (b) original test set separability of DCE sequences; (c) test set separability after SMMF framework classification; blue dots represent HGUC and orange dots represent LGUC.


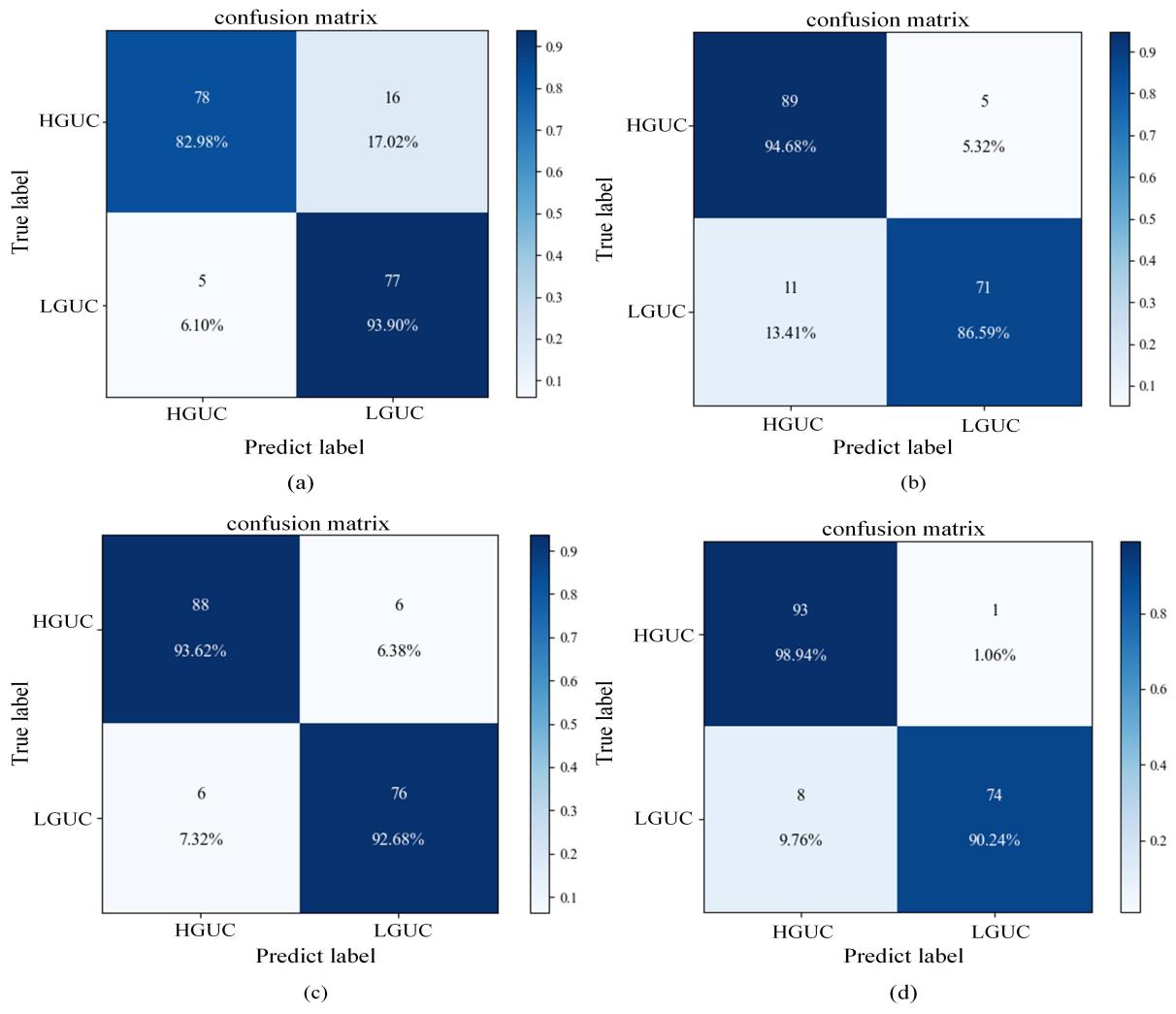


**Supplementary Figure 7.** Confusion matrix of different feature fusion strategies. (a) Input-level fusion; (b) Feature-level fusion; (c) Decision-level fusion; (d) SAFF.


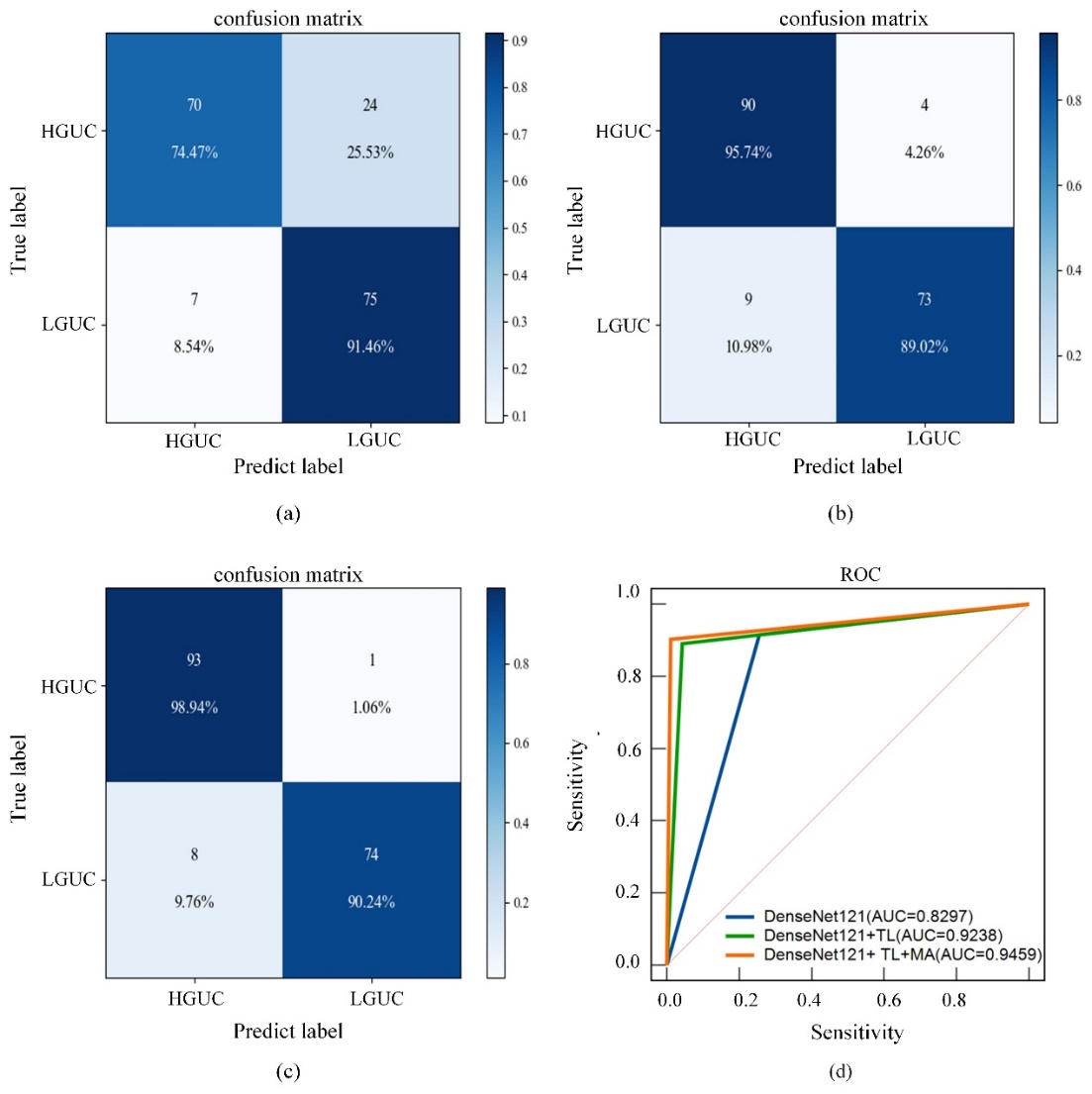


**Supplementary Figure 8.** SMMF framework. CNN: extraction of underlying BCa features; multi-scale attention model (MA): extraction of rich multi-scale features; self-attention feature fusion model (SAFF): fusion of MP-MRI features.
